# Artemin的研究进展

**DOI:** 10.3779/j.issn.1009-3419.2011.10.05

**Published:** 2011-10-20

**Authors:** 广苏 寻, 志刚 李

**Affiliations:** 1 450052 郑州，郑州大学第一附属医院胸外科 Department of Thoracic Surgery, the First Affiliated Hospital of Zhengzhou University, Zhengzhou 450052, China; 2 天津医科大学总医院，天津市肺癌研究所 Tianjin Lung Cancer Institute, Tianjin Medical University General Hospital, Tianjin 300052, China

尽管肺癌外科治疗技术和多学科综合治疗等有了很大的进步，肺癌总的治愈率仍仅为10%-15%，而I期肺癌的5年生存率可达80%以上。其主要原因是缺乏有效的早期诊断手段和治疗肿瘤转移的有效方法。恶性肿瘤的发生、发展、侵袭和转移是一个极其复杂的多阶段、多步骤的过程，早期诊断和早期治疗是提高患者生存率和降低死亡率的关键^[1]^。Artemin（ARTN）属于胶质细胞源性神经营养因子配基家族，可介导各种类型神经元的存活、分化和迁移。ARTN及其受体（GFRα3/RET）在恶性肿瘤细胞中表达升高，并参与了各种恶性肿瘤的进展^[2]^。对ARTN的临床研究或许能对肿瘤的早期诊断和早期治疗提供帮助；对其信号转导和功能性研究或将为恶性肿瘤的靶向治疗开辟道路。

## 1 ARTN的分子结构

GDNF（glial cell line-derived neurotrophic factor）、NTN（neurturin）和PSP（persephin）被发现后，研究者试图寻找GDNF家族的其它成员。1998年12月Baloh等^[3]^发现了GDNF家族的第4个成员。他们以成熟NTN蛋白顺序为标准，通过高含量基因组顺序（high throughput genome sequences, HTGS）数据库发现两个细菌人工染色体（bacterial artificial chromosome, BAC）克隆含有与GDNF、NTN及PSP相似但不相同的同源开放阅读框（homologous open reading frame, hORF），表达序列标签（expressed sequence tag, EST）数据库表明该hORF为一种表达基因，利用PCR方法也扩增到与该hORF相关的鼠基因组DNA，并利用cDNA末端的随机扩增（random amplification of cDNA ends, RACE）技术从人及鼠的组织cDNA文库扩增到全长的cDNA顺序，经分析全长cDNA，表明hORF编码的是一种新的GDNF家族成员，即ARTN。人ARTN基因有3个外显子和2个内含子，位于染色体1p32-33区，ARTN蛋白一级结构与GDNF、NTN、PSP相似，相对位置具有相同的7个保守的半胱氨酸残基，全长约3.9 kb，经不同剪切形成4种mRNA，其大小分别为1, 408 bp、1, 892 bp、1, 162 bp和1, 161 bp。人ARTN基因开放阅读框分别编码237个、228个及220个氨基酸的前体蛋白，前体蛋白N端为信号肽，由39个氨基酸组成，其后为前体区，在202位存在1个N-糖基化位点（NSTW），在RXXR（RAAR）识别位点处经蛋白酶切割后加工成113个氨基酸序列的成熟ARTN蛋白。其氨基酸顺序与NTN和PSP更接近，约45%同源，与GDNF同源性约36%。

## 2 ARTN的表达分布

成年和发育中的大鼠，其纹状体、皮层、小脑、海马和脊髓中都有ARTN少量存在；在成人的纹状体、皮层、海马和脊髓中也检测到ARTN mRNA；另外，新生大鼠中枢神经系统以外的组织如肾、肺、心、脾、肝、血液、坐骨神经、睾丸、卵巢、皮肤、骨骼肌、肾上腺都有ARTN mRNA微量表达。ARTN在体内广泛分布，提示它们对于神经系统和非神经组织的发育及生理功能的维持具有重要意义^[4, 5]^。

ARTN mRNA在体内分布广泛，ARTN mRNA分布介于GDNF、NTN与PSP之间。体外试验^[6]^表明，成人外周组织中垂体、胎盘、气管高水平表达，在胎儿中只有肾、肺高水平表达；成人及胎儿脑组织，包括基本神经节（下丘脑核、豆状核、黑质）及丘脑表达水平都很低，提示ARTN可能作用于皮层下运动系统；脊髓及背根神经节表达量也低，经过14天的大鼠胚胎（E14）的进一步研究表明，在神经根（不包括背根神经节）及肠系膜上动脉周围高表达，提示ARTN对于外周神经系统的作用可能有两种方式：对于背根神经节发育中的神经元以旁分泌方式起作用和对肠系膜上动脉等自主神经支配组织起到靶源性因子的作用。

ARTN在人类的各种正常组织和相应组织来源的恶性肿瘤中都有分布，并且呈差异性分布。应用Oncomine数据库分析人类不同组织来源的癌细胞中ARTN的表达水平，并且与其组织来源相同的正常组织中的ARTN表达水平相比，研究^[2]^发现，在一些常见癌肿（包括肺癌、卵巢癌、宫颈癌、胰腺癌、皮肤癌、头颈癌、精原细胞瘤、白血病）中ARTN表达升高，总体趋势是恶性肿瘤组织比相应正常组织高表达。

## 3 ARTN的信号转导

ARTN是分泌性蛋白，这决定了其对细胞的营养作用必须通过跨膜信息传递作用即受体介导才能实现。复合受体由两部分组成，第一部分是糖基化的磷脂酰肌醇（glycosyl-phosphatidylinositol, GPI）锚定到细胞表面的蛋白分子，称为GDNF家族受体α（GDNF family receptor alpha, GFRα）直接与GDNF结合；另一部分为原癌基因c-ret编码的产物蛋白Ret，它是一种跨膜的受体酪氨酸激酶。前者特异性地结合GDNF家族成员，促使Ret磷酸化，磷酸化的Ret激活其下游的丝裂原活化蛋白激酶（mitogen-activated protein kinase, MAPK）等，导致一系列胞内途径的激活，从而发挥GDNF家族神经营养因子的生理功能^[7, 8]^。GDNF家族各成员的Ret是一样的，但GFRα有所不同，其特异性是由前者决定的^[9]^。

### 3.1 GDNF家族受体

目前的研究表明GFRa至少有4种，即GFRα1、GFRα2、GFRα3和GFRα4。Jing^[10]^用^125^I标记的GDNF筛选新生大鼠视网膜细胞的cDNA表达文库，得到1个2, 138核苷酸的cDNA片段，从302核苷酸处开始编码468氨基酸的多肽，命名为GFRα。多肽的N-疏水端有19个氨基酸的分泌性信号肽，C-端有20个氨基酸的疏水区，由于缺乏亲水结构使其与膜磷酯酰肌醇相偶联。用放射性标记的大鼠GFRα cDNA筛选人黑质cDNA文库，获得了人GFRα cDNA其编码的多肽长465氨基酸，与大鼠有93%的同源性。GFRα3是ARTN的受体，其表达与GFRα1和GFRα2不同，表达部位有严格限制性，主要见于外周神经系统如背根节、三叉神经节、交感节和神经纤维鞘细胞以及非神经元细胞（肾上腺嗜铬细胞、肠上皮细胞）等，中枢神经系统中未见表达^[11]^。而GFRα1和GFRα2广泛表达于中枢神经系统和外周器官^[11, 12]^。GFL结合到特异性的GFRα决定了受体复合物的信号特异性，ARTN优先结合于GFRα3，但这种结合的特异性并不是绝对的，如ARTN可以和GFRα1结合^[13]^。

### 3.2 Ret-复合受体

由于GFRα是GPI连接的胞外蛋白，缺乏跨膜区及膜内区，无法单独完成信号传导。神经营养因子与GFRα特异结合之后，尚需一种跨膜蛋白的介导，信号才能进一步传入细胞内，这种跨膜蛋白即Ret蛋白，它与GFRα协同作用，共同参与GDNF家族神经营养因子的信号传导。原癌基因*c-ret*编码的产物Ret蛋白是一种受体酪氨酸激酶，它与GFRa共同构成GDNF家族神经营养因子的功能性复合受体。Ret分子最初是作为一种癌基因被发现的，其激活性突变与人甲状腺癌，II型家族性内分泌瘤胞（MENII）的发生相关，失活性突变则引起Hirchsprung病（无神经结性巨结肠）。Ret分子的胞外有钙粘附素（Cadherin）样结构、半胱氨酸富集区，胞内有酪氨酸激酶区，有14个酪氨酸残基，其中第905位、1, 015位、1, 062位、1, 096位为磷酸化的酪氨酸残基，分别为生长因子受体结合蛋白（Grb）7/10、PLC、Shc/Enigma、Grb2等蛋白结合位点，其中PLC通过IP3引起胞内钙释放，其它则参与Ras-MAPK-CERB信息通路，Grb2还可能参与PI3K-AKt活动^[7]^。NBL-S细胞系可表达Ret，与ARTN作用后，Ret酪氨酸磷酸化水平升高，用PIPLC处理后，则ARTN引起的Ret磷酸化水平明显下降，加入可溶性的GFRα3后，又明显升高；免疫沉淀及Western blot进一步表明，GFRα与Ret在细胞表面形成一个功能性的受体复合物，前者特异性地结合GDNF家族成员，后者进行跨膜信息转导。可见，GDNF家族神经营养因子结合GFRα后，导致Ret酪氨酸激酶磷酸化，磷酸化的Ret引起下游激酶如MAPK、PI-3激酶等的激活，从而将信息传递到细胞内，发挥GDNF家族成员的生理功能^[3, 7, 14]^。

## 4 ARTN在神经系统中的生理功能

ARTN对多种神经元的存活和分化起作用，ARTN可促进培养的中脑多巴胺能神经元形态分化，在多巴胺能神经元结构发育和可塑性中起到重要作用^[15, 16]^。应用Western blot技术和免疫组织化学方法对胎儿及成人组织进行检测，结果表明ARTN可促进成人海马神经元的存活，并对维持其功能起作用^[17]^。在体外培养中，经ARTN处理的颈动脉体血管球细胞的数量增多、胞体增长，这说明ARTN对颈动脉体的功能起作用，并有可能通过颈动脉体转运提高其神经定向分布^[18]^。通过对11.5日孕龄（E11.5）到出生后7日（P7）小鼠的研究^[19]^发现，ARTN在交感神经节神经元轴突发育早期阶段对诱导突触生长起作用，而GDNF和NTN在发育较晚阶段对交感神经节神经元轴突定向生长有重要作用。ARTN还可对交感神经元分化、存活和生长起作用，促进E12到E14交感神经元的分化^[20]^。ARTN对培养的大鼠中脑GABA能神经元和血清素激活神经元也具有明显作用^[21]^。虽然在大鼠胚胎中脑腹侧未检测到ARTN表达，但经体外培养的多巴能神经元试验表明，ARTN也能促进多巴胺能神经元的存活^[22]^。

ARTN、GDNF和NTN在体外均能促进多种发育中的外周神经元包括交感神经元、副交感神经元及感觉神经元的存活。首先，对颈上神经节交感神经元具有营养与支持作用；其次，也能促进多种神经元如背根神经节、三叉神经节感觉神经元存活，在这两群感觉神经元中，ARTN促进存活的神经元数量比GDNF、NTN多；另外，对鼻根神经节、内脏感觉神经元的促进存活能力相当。而PSP对于外周神经系统不起作用^[3, 6, 23]^。

ARTN能促进运动神经元的存活。腰段脊髓切面乳剂放射自显影技术显示，大鼠坐骨神经的运动神经元轴突能以特异的受体介导方式逆向运转ARTN到神经元胞体，实验证实，GDNF、NTN和PSP也能以同样的方式运转到神经元胞体，这些结果说明GDNF家族成员对运动神经元具有作用^[24]^。实验结果表明PSP对运动神经元的存活作用比ARTN、GDNF、NTN等三个因子作用要小得多。

## 5 ARTN在肿瘤中的研究进展

### 5.1 ARTN与乳腺癌

ARTN在人类乳腺癌中的表达可使肿瘤细胞恶性程度增加^[25]^。ARTN广泛表达于人类乳腺癌细胞系中，上调ARTN在乳腺癌细胞中的表达可将提高乳腺癌细胞的非贴壁生长，增加软琼脂和三维基质胶中肿瘤细胞群落的形成数量；促进形成分散生长的肿瘤细胞的表型，增加乳腺癌细胞的转移和侵袭特性。上调ARTN的表达水平还将增加肿瘤的大小。在异种移植模型中发现，上调ARTN的表达水平将强化高增殖、低分化和高侵袭性的乳腺癌肿瘤细胞的恶性特性。Oncomine^[2]^数据库分析显示，较高的ARTN的表达水平与乳腺癌化疗后残余癌灶的转移、复发、死亡呈正相关。ARTN蛋白在65%的乳腺癌患者中表达，它的表达明显降低了这一类患者的总体生存率；通过人为干涉ARTN mRNA的转录过程或用其抗体阻断其在乳腺癌细胞的内源性表达和拮抗其生理功能，将降低乳腺癌细胞的转移性和侵袭性。可见，ARTN在人类乳腺癌细胞中具有致癌源性，因此抑制ARTN的生理功能是乳腺癌靶向治疗中应当考虑的因素。

*ARTN*是一个雌激素诱导基因，基因数据库的表达分析显示，在人乳腺癌中*ARTN*的基因表达与雌激素受体的表达呈正相关^[26]^。在雌激素受体阳性的乳腺癌患者中，ARTN的高表达明显降低群体生存率。在雌激素受体阳性的乳腺癌细胞中，上调ARTN的表达将促进雌激素受体的转录，提高雌激素的表达水平，进一步增强对他莫西芬和氟维斯群的药物抵抗。ARTN的表达增强细胞对他莫昔芬和氟维斯群的抵抗是通过增加bcl-2的表达来实现的。反之，通过阻断ARTN mRNA的表达或通过ARTN的抗体阻断ARTN的功能，则增加了雌激素抵抗效力。在对他莫昔芬敏感的乳腺癌细胞中，他莫昔芬降低了ARTN的表达；同样在他莫昔芬抵抗的乳腺癌细胞中，ARTN的表达升高。用抗体抑制ARTN表达可提高对他莫昔芬抵抗的乳腺癌细胞对他莫昔芬的敏感性，因此在乳腺癌细胞中功能性拮抗ARTN的功能可作为一个辅助治疗去强化雌激素抵抗效应。

虽然ARTN的表达水平在正常组织和乳腺癌组织中差异不大，有研究^[27]^发现在乳腺癌中ARTN明显比在其它类型的癌中高表达（*P* < 0.001）；从不典型导管增生到乳腺癌的形成过程中，ARTN的表达逐渐升高（*P* < 0.042）；ARTN在乳腺基底瘤比顶质瘤和网状瘤高表达^[28]^；在侵袭性导管癌比乳腺原位导管癌高表达^[29]^；乳腺癌从Ⅰ期到Ⅲ期ARTN的表达逐渐升高^[30]^（r=0.274）；另外ARTN的表达与雌二醇受体和孕体酮受体正相关。三份独立研究报告^[31-33]^表明乳腺癌雌二醇受体阳性的比阴性的ARTN高表达；二份独立研究报告^[33, 34]^显示乳腺癌孕体酮受体阳性比阴性细胞的ARTN表达高；男性乳腺癌细胞比女性乳腺癌细胞的ARTN表达高^[35]^；HER2/neu（c-erbB-2）阳性的乳腺癌肿瘤细胞比阴性细胞的ARTN表达低^[31, 32]^。

ARTN的表达升高多提示乳腺癌的复发或预后不良。一份研究报告^[36]^显示乳腺癌复发并雌二醇受体阳性的患者明显比生存长于5年的患者ARTN表达高；二份研究报告^[37, 38]^显示预后不良的患者比有生存长于5年的患者ARTN高表达；患者有远处转移的明显比没有远处转移的ARTN高度表达^[38]^；对化疗敏感的患者明显比化疗不敏感的患者ARTN低表达（*P* < 0.001）；所有患者^[31, 38]^中乳腺癌雌二醇受体阳性比阴性ARTN高表达（*P* < 0.005）；因此ARTN在乳腺癌中的表达水平与患者的预后有明显的相关性。GDNF的表达谱在人类各种癌症和正常组织中的对比分析也被Oncomine数据库分析，相比ARTN，其缺乏疾病与预后的相关性。

有免疫组织化学分析法对ARTN在乳腺癌中的表达情况分析示^[25]^：乳腺癌细胞中ARTN比正常乳腺组织细胞高表达（*P* < 0.001），ARTN的表达高低与年龄、肿瘤大小、淋巴结有无转移、临床分期、雌激素受体状态和孕酮受体状态无关，与HER2/neu（c-erbB-2）的表达高低呈正相关。

### 5.2 ARTN与胰腺癌

胰腺导管腺癌以局部神经改变和外周神经从侵袭著称，导致严重疼痛和妨碍完整切除癌肿手术的完成。ARTN神经营养蛋白控制神经元生长、再生、存活，可用于分析胰腺导管腺癌和神经转移之间的相互关系。用蛋白印记分析和免疫组织化学分析对ARTN和它的受体（GFRα3/RET）在胰腺导管腺癌和正常胰腺中的表达情况分析^[39]^：ARTN及其受体在胰腺导管腺癌中比正常胰腺组织中ARTN表达高，主要集中在肥大的神经、动脉壁、原位癌灶和转移癌灶中。通过定量逆转录PCR对胰腺癌组织、正常胰腺组织和胰腺癌细胞系中ARTN mRNA的表达分析，去评估ARTN是否影响癌细胞的增殖和侵袭：ARTN高表达提升胰腺癌细胞的侵袭能力近5倍，ARTN是趋神经因子似乎增进胰腺癌沿神经的侵袭，也促进了癌细胞沿着胰腺神经向远处转移。MTT生长分析和三维基质胶实验被应用以评估癌细胞增殖情况与ARTN表达的关系，结果示ARTN的表达高低对癌细胞的增殖没有影响。在胰腺导管腺癌中组织的ARTN表达水平与患者的疼痛并不相关。

有研究^[40]^采用免疫组化和RT-PCR方法检测胰腺癌组织、癌旁组织和正常胰腺组织中ARTN GFRα3的表达，结果显示胰腺癌组织中ARTN、GFRα3阳性表达率分别为72.09%和67.44%，均明显高于癌旁组织（*P* < 0.01），癌旁组织中ARTN、GFRα3仅出现在动脉平滑肌细胞中，在胰腺内神经丛和导管未见明显表达。在胰腺癌组织中ARTN、GFRα3在神经、癌细胞中均有较强表达：ARTN、GFRα3在有神经浸润的胰腺癌组织中阳性表达率明显高于无神经浸润者，其差异有统计学意义。以上证明了ARTN、GFRα3与胰腺癌细胞的生物学行为有关，他们之间的相互作用可能促进了胰腺癌细胞侵袭能力，直接导致癌细胞侵至胰腺内外神经丛，这与Veit等^[41]^的结果一致。有研究^[42]^也显示，胰腺癌中ARTN mRNA明显高于正常胰腺组织，有神经浸润的胰腺癌组织也明显高于无神经浸润的胰腺癌组织，但ARTN mRNA增高的水平远低于蛋白的增高水平，可能是因为胰腺外神经丛产生的ARTN蛋白也聚集于胰腺内神经丛。密集的神经网络和肥大的神经不仅在胰腺癌中存在还在正常的癌旁组织中存在，而在正常胰腺中很少见或不常见。但是在组织学正常的胰腺癌旁组织或胰腺癌中的出现可能与胰腺内的神经转移密切相关。利用胰腺癌、癌旁组织或胰腺癌细胞株悬浮提取物培养肠肌神经丛明显增加了神经突的数量。当去除ARTN后培养，神经突的密度明显降低^[42]^。看似正常的癌旁组织很可能已通过神经趋化因子转移，尽管没有证实神经与癌的交互作用导致了微转移。胰腺癌的神经介导转移作用可以在体外建模模拟，进一步揭示了ARTN很可能在胰腺癌的神经转移中扮演重要角色。

有资料^[3]^显示，ARTN蛋白主要产生于背根神经节。癌细胞持续损伤神经纤维，促进背根神经元产生大量ARTN蛋白，用于修复保持被损伤神经纤维的完整性，ARTN蛋白沿神经轴突逆向输送至胰腺内神经丛，导致胰腺内ARTN蛋白的表达明显高于其mRNA表达水平。总之，ARTN和GFRα3在胰腺癌中的表达及其相互作用能诱导和促进胰腺癌神经浸润转移，这将有助于进一步明确胰腺癌神经浸润转移的发生机制。同时可能通过对ARTN、GFRα3的表达调控或基因转染方法，与其它化疗、放疗等方法共同干预胰腺癌细胞内环境和宿主微环境，达到抑制胰腺癌浸润转移、改善胰腺癌预后的目的。

### 5.3 ARTN与食管癌

有研究^[43]^结果显示ARTN在食管癌中的表达水平明显高于正常食管组织，而且在不同临床病理分期之间表达差异有统计学意义（*P* < 0.01），实验结果表明，在食管癌中随着疾病的进展，ARTN阳性表达率也逐渐升高，在发生淋巴结转移的食管癌中ARTN的阳性表达率也明显高于未发生淋巴结转移的食管癌组织（*P* < 0.01），提示ARTN的阳性表达可能与食管癌的淋巴结转移相关。

基质金属蛋白酶2（matrix metalloproteinase-2, MMP2）是一类内肽酶，能够降解细胞外基质成分，生理状态下参与组织的重构和伤口的愈合。在肿瘤的形成过程中能够降解基底膜而促进肿瘤的侵袭、转移，还能够促进毛细血管的生成，促进肿瘤的生长和扩散^[43, 44]^。O-charoenrat等^[45]^研究发现MMP2的激活能够通过p53介导的SCCRO引起肿瘤细胞的浸润和转移。有研究^[43]^表明MMP2的表达与ARTN呈正相关，ARTN与MMP2同时阳性表达的病例数明显高于两者同时阴性表达的病例数，数据统计分析显示ARTN与MMP2之间存在明显的相关性。李红梅等^[46]^的研究结果显示：转移能力相对较高的细胞系产生的MMPs的能力强于转移能力相对较低的细胞系，两项研究结果相一致。结果显示两者的联合表达可能与食管癌的疾病进展、侵袭和远处转移有密切的联系。Kolb等^[47]^研究发现重组表达ARTN能促进癌细胞的浸润，利用RNA干扰剂阻断ARTN的表达后，胰腺癌细胞的浸润能力下降，在食管癌中的研究目前未见报道，进一步研究ARTN在肿瘤发生发展中的作用，将有助于我们设计合理的靶向治疗药物，对食管癌的生物治疗提供理论基础。

### 5.4 ARTN与子宫内膜癌

已有研究^[48]^证实在对子宫内膜癌用阿霉素和紫杉醇化疗时，联合应用ARTN的功能性抗体，将降低子宫内膜癌细胞的增殖性和侵袭性。因此，针对ARTN的靶向治疗或许是子宫内膜癌很有前景的治疗方法。目前，已经证实在子宫内膜癌中CD24是经过介导ARTN的功能来抵抗阿霉素和紫杉醇的，很有可能CD24是启动相关基因的中间产物。在RL95-2细胞株ARTN和CD24均能启动相关基因，如*CCND1*、*BCL2*、*AKT1*、*TWIST1*和*VIM*，并降低BAD和PLAU的mRNA表达。功能性抑制ARTN的功能，将增加子宫内膜癌对阿霉素和紫杉醇的敏感性。因此，用ARTN的功能性抑制抗体拮抗其功能，对子宫内膜癌的治疗是有效的，无论是抑制肿瘤细胞的侵袭性还是提高其对化疗药的敏感性。在子宫内膜癌中ARTN通过上调CD24的转录增加其表达，因此它们的表达具有相关性。在子宫内膜癌CD24的高表达将增加癌细胞的增殖、侵袭和降低对阿霉素和紫杉醇的敏感性。拮抗CD24的表达将消除经过ARTN激活的对阿霉素和紫杉醇的抵抗。因此，在子宫内膜癌中ARTN的高表达将提升对阿霉素和紫杉醇的抵抗，这是通过CD24的特殊调节过程来实现的。功能性抑制ARTN和CD24的表达可以被认为是一个有效的辅助治疗，来增加子宫内膜癌对化疗药的敏感性。

在子宫内膜癌中ARTN的表达量比正常子宫内膜组织明显增加，并且ARTN的表达水平与子宫内膜癌的分期和侵袭性呈正相关。在子宫内膜癌细胞中增加ARTN的表达可以缩短细胞循环周期和提高细胞的存活率，因此可以明显增加总的细胞数量。此外，上调ARTN的表达明显增加子宫内膜癌细胞的非贴壁生长。在上调ARTN移植模型中，癌细胞表现出高增殖、低分化、高侵袭的特性。ARTN的表达上调增加致癌性和侵袭性是通过增加AKT1的表达来实现的^[49]^，在子宫内膜癌细胞中对ARTN mRNA的阻断和用抗体对其的功能性阻断都将明显降低其癌源性和侵袭性。因此，阻断*ARTN*基因转录过程和拮抗其生理功能将成为子宫内膜癌的治疗策略，以延缓其进展。

### 5.5 ARTN与肺癌

#### 5.5.1 ARTN及其受体在人类肺癌中的表达情况

Oncomin数据库^[2]^对人类的肺癌细胞基因的表达进行研究，发现ARTN、GFRA1-A3和RET在正常肺组织和肺肿瘤细胞中的表达存在差异。为了检测ARTN在肺癌中的表达情况，本实验室对载有腺癌35例、鳞癌33例、大细胞癌9例、小细胞癌12例和正常肺组织10例的组织芯片进行免疫组化分析，以检测ARTN在不同肺癌组织中的表达情况（图 1）。免疫组化示ARTN在正常肺组织、非小细胞肺癌和小细胞肺癌中都有不同程度的表达；相比于正常的肺组织，ARTN在类癌、非小细胞肺癌（腺癌和鳞状细胞癌）和小细胞癌中的表达水平明显升高。ARTN的高表达与鳞状细胞癌细胞去分化密切相关，可以提高肺部鳞状细胞癌的临床分期，促进鳞状细胞癌的进展。相对于肺癌的其它亚型来说，ARTN在鳞状细胞癌中表达最高。Bild^[33]^曾指出：ARTN在鳞状细胞癌中的表达水平比在腺癌中高出96%。GFRA1在肺癌各个亚型中的表达水平是不一样的，还没有准确的证据说明GFRA1的高表达能促进腺癌进展。相比于正常肺组织，GFRA2在恶性肿瘤（除类癌）的表达水平明显降低，GFRA2在鳞状细胞癌中表达预示着高分化、鳞状细胞肺癌早期。GFRA3的表达水平在类癌、腺癌、鳞癌和小细胞肺癌中明显比正常肺组织中高；Ret的表达水平在腺癌中明显比在人类正常肺组织中高。相对于肺癌的其它亚型（鳞癌、大细胞癌、小细胞癌）来说，腺癌细胞RET明显高表达。

**1:**
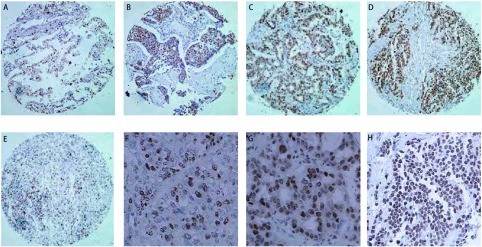
免疫组化法检测肺癌组织中ARTN的表达。A：正常肺组织（×100）；B：鳞癌（×100）；C：腺癌（×100）；D：小细胞肺癌（×100）；E：大细胞肺癌（×100）；F：鳞癌（×200）；G：腺癌（×200）和H：小细胞肺癌（×200） The status of ARTN expression in lung cancer cells by immunohistochemistry. A: normal lung tissue (×100); B: squamous cell carcinoma (×100); C: adenocarcinoma (×100); D: small cell lung carcinoma (×100); E: large cell lung carcinoma (×100); F: squamous cell carcinoma (×200); G: adenocarcinoma (×200); H: small cell lung carcinoma (×200)

我们采用半定量RT-PCR和Western blot分别对8株非小细胞肺癌细胞株NL9980、L9981、95D、LTEP-α-2、SPCA-1、YTLMC-9、PC-9和A549进行检测，结果如（图 2A、图 2B）所示。半定量RT-PCR和Western blot示：ARTN在细胞株LTEP-α-2、SPCA-1、YTLMC-9、PC-9比在细胞株NL9980、L9981高表达。另外，有研究^[50]^显示：ARTN、GFRA3和RET的RNA在非小细胞肺癌细胞株H1299、H1975、H460、H2009和A549细胞系中均检测表达，它们的表达水平不同；GFRA成员在各个细胞系中表达差异很大：GFRA1仅在H1299、H460和A549细胞系中检测到，GFRA2却在H1975和A549细胞系中检测到。

**2:**
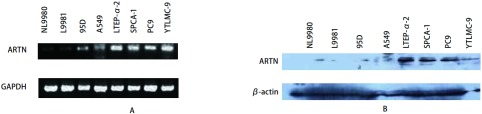
RT-PCR/Western blot检测ARTN mRNA（A）/蛋白（B）在9种肺癌细胞株中的表达 The relative express level of ARTN expression in eight NSCLC cell lines by RT-PCR (A)/Western blot (B)

#### 5.5.2 ARTN的高表达提高H1299细胞的增殖、转移和侵袭能力

Tang^[50]^转染了非小细胞肺癌细胞系H1299，用一个编码人类ARTN基因的（pIRESneo3-ARTN）质粒和一个空的媒介质粒pIRESneo3，分别产生了稳定H1299-ARTN和H1299-VEC细胞系，并用实验证实H1299-ARTN细胞中ARTN的表达水平明显高于对照组H1299-VEC细胞。在10%胎牛血清培养基中单层培养实验中，没有观察到H1299-VEC和H1299-ARTN细胞数目存在差异。定量PCR分析也没能发现因相关基因改变细胞周期而导致的增殖差异。然而，他们观察到在低血清的培养基（0.2% FBS）中，H1299-ARTN的细胞数目比H1299-VEC细胞数目多48%。PI染色和流式细胞仪分析并未发现H1299-VEC和H1299-ARTN细胞在细胞周期各阶段分布情况中存在明显不同，只是观察到有不同的细胞总数，或许归因于不同细胞的固有凋亡比率。在紫杉醇用于治疗非小细胞肺癌的临床用药试验中^[51]^，通过紫杉醇诱导H1299-细胞凋亡模型，流式细胞仪分析和PI染色分析测定H1299-ARTN的早期、晚期和总体细胞凋亡率分别为48%、57%和54%，明显低于H1299-VEC细胞。

Tang^[50]^检测了ARTN高表达对H1299非贴壁生长的影响，对于一种转化的细胞表型，这是一个特征性生理标志。在软琼脂上H1299-ARTN形成的细胞集落比H1299-VEC细胞多出91%。在三维基质胶培养中H1299-ARTN的细胞数比H1299-VEC多61%。在细胞悬浮培养实验中，H1299-ARTN的细胞数目比H1299-VEC多82%。在三维基质胶中，H1299-ARTN形成的球形细胞群落具有星状散射的形态，并且明显比H1299-VEC多。H1299-ARTN在三维基质胶中表现出较强的生长能力和扩散形态，因此ARTN很可能提高了非小细胞肺癌的侵袭能力。

在转移性分析中，H1299-ARTN细胞株的转移细胞比H1299-VEC细胞多131%。在划痕试验中H1299-ARTN细胞明显比H1299-VEC细胞修复划痕的速度快。在侵袭性分析中，H1299-ARTN细胞通过转孔侵袭的数目比H1299-VEC细胞多128%。定量PCR显示，TGFB1在H1299-ARTN细胞中的表达水平明显比H1299-VEC细胞升高，被认为是非小细胞肺癌的侵袭刺激因子^[52]^。

#### 5.5.3 在异种移植模型中ARTN高表达增强了肿瘤细胞的恶性特性

用H1299-ARTN细胞构建的老鼠异种移植肿瘤模型，检测在模型中ARTN的高表达对肿瘤细胞特性的影响^[50]^。用皮下注射的方法将H1299-ARTN细胞和H1299-VEC细胞分别注射到有免疫缺陷的老鼠的肩胛下区域，第30天，H1299-ARTN细胞衍生肿瘤比H1299-VEC细胞衍生的肿瘤明显增大，增大比例约63%。他们通过检测肿瘤细胞的溴脱氧尿苷来计数细胞进入S期的比率，合用TUNEL染色在肿瘤的每个部分检测肿瘤细胞的凋亡情况，检测结果显示：H1299-ARTN细胞衍生肿瘤进入S期的细胞比H1299-VEC细胞衍生肿瘤多45%，而凋亡比率则降低了37%。组织病理学检测发现H1299-VEC细胞衍生的肿瘤是有规则的肿瘤细胞形成的局限性肿瘤，它们与周围组织有着清晰的分界。然而H1299-ARTN细胞衍生的肿瘤是较松散的肿块，有些地方有空泡形成，这些区域聚集很多大的多形细胞，这些细胞表现出形态极不规则，具有多重深染的细胞核和大部分细胞质，与周围组织边界不清，并表现出粘蛋白的特征。同时还观察到，H1299-ARTN衍生肿瘤细胞还向神经纤维靠近生长，预示着具有外周神经侵袭特性。在H1299-ARTN衍生肿瘤所在老鼠的肺中，还发现转移结节（2/6），但是并没有在H1299-VEC细胞衍生肿瘤所在的老鼠中发现（0/6）。Tang^[50]^同样转染了非小细胞肺癌细胞系H1975，分别产生了稳定H1975-ARTN和H1975-VEC细胞系。ARTN的高表达同样提高了H1975细胞存活率、转移和侵袭能力。因此，ARTN在非小细胞肺癌中的高表达预示着肿瘤细胞具有更高的增殖和存活率，并由此引发肿瘤的快速生长和高侵袭，并最终导致远处转移。

#### 5.5.4 ARTN经过BCL2介导增强非小细胞肺癌细胞的恶性程度

研究^[50]^发现抗细胞凋亡因子BCL2 mRNA表达水平在高表达ARTN的肿瘤细胞中明显升高。与其对应的对照组H1299-VEC/H1975-VEC相比，在H1299-ARTN细胞中BCL2 mRNA表达水平升高165%；H1975-ARTN细胞BCL2 mRNA的水平升高356%。此外，他们还发现高表达ARTN可激发BCL2启动子活性：H1299-ARTN细胞BCL2启动子活性比H1299-VEC细胞高49%；同时H1975-ARTN细胞BCL2启动子活性比H1975-VEC细胞高383%。Western blot分析结果与以上是一致的；进一步揭示了H1299-ARTN和H1975-ARTN细胞中的BCL2蛋白表达水平均比它们相应的对照组增高。他们把H1299-VEC和H1299-ARTN细胞经过BCL2抑制剂YC137^[53]^处理后，在数量和形态上评估上述细胞在软琼脂和三维基质胶的生理状态。在H1299-VEC细胞中YC137产生一个剂量依赖性降低软琼脂集落形成的作用。YC137可以明显消除由ARTN刺激强化的H1299-ARTN细胞的非贴壁生长，这些都证明ARTN在软琼脂中促进细胞集落的形成是依赖BCL2介导的。此外，YC137明显地降低了H1299-ARTN细胞由ARTN刺激引起的在三维基质胶中的生长速度，即使在H1299-VEC细胞中，通过抑制BCL2的表达也一样降低了该细胞的生长速度。

#### 5.5.5 抑制或阻断ARTN可降低非小细胞肺癌细胞恶性程度

有研究^[50]^通过小干扰RNA内源性拮抗ARTN在两个非小细胞肺癌细胞系的表达，并检测其对细胞功能影响结果。他们构建转染筛选了稳定的H1299-siARTN/H1299-CONTROL和H1975-siARTN/H1975-CONTROL细胞系，靶向拮抗了ARTN的内源性表达，同时与相应阴性对照组对比研究。半定量RT-PCR和Western blot分析显示，H1299-siARTN和H1975-siARTN细胞株的ARTN mRNA和ARTN蛋白的表达明显比它们相应的对照组H1299-CONTROL和H1975-CONTROL细胞株降低。H1299-siARTN细胞与H1299-CONTROL细胞比较：降低ARTN表达的H1299-siARTN细胞降低32%细胞集落形成，降低54%细胞转移，降低49%细胞侵袭，降低31%在三维基质胶中的生长速度。同样，相似的结论在H1975-siARTN细胞和H1975-CONTROL细胞比较中得到。他们在非小细胞肺癌细胞系中用抗ARTN抗体功能性抑制ARTN的作用，进一步评价ARTN的生理功能^[50]^。把H1299细胞和H1975细胞系分别用鸡ARTN单克隆抗体（ARTN-IgY）处理，并将它们设为实验组。用预免疫的鸡单克隆抗体（CON-IgY）处理的细胞作为相应的阴性对照组；用软琼脂集落试验和三维基质胶生长试验来检测两组细胞生理功能的差异。用ARTN-IgY处理过的H1299细胞，在软琼脂中的集落比对照组明显缩小23%；在三维基质胶生长试验中这些差别得到了重现。另外，H1299细胞经过ARTN-IgY处理后，该细胞相比于对照组细胞转移性降低45%，侵袭能力降低48%。在H1975细胞经过ARTN-IgY处理后，与对照组H1975ARTN-CON-IgY相比，在软琼脂和三维基质胶中的生长、侵袭和转移能力均有不同程度的降低。总体来说，细胞存活率也降低，有相当大比例的H1975-ARTN-IgY细胞在软琼脂和三维基质胶生长试验中停止生长，并渐渐消失。

#### 5.5.6 ARTN受体表达情况对肺癌细胞生理特性的影响

ARTN在H1299肺癌细胞株中的表达相对于肺癌细胞株H1975来说并不高。然而在肺癌细胞株H1299和肺癌细胞株H1975中去除或抑制ARTN的表达对该细胞生理功能的影响是相似的。一些特殊细胞对配体的反应并不单纯取决于配体的浓度，如一些配体完全激动受体信号环路仅仅只需占据5%的受体^[54]^。还需要指出H1299表达GFRα3和GFRα1两种受体，而H1975细胞株仅仅表达GFRα3受体。ARTN已被证实刺激GFRα1-RET复合体的形成，这是需要GFRα3受体参与的^[55]^，因此H1299细胞株同时有GFRα3和GFRα1两种受体。相对于只能表达GFRα3一种受体的H1975细胞株，在ARTN较低浓度可以对各种功能反应有良好的应答。胶质细胞源性神经营养因子配体已被证实可以结合非GFRα蛋白^[56, 57]^，功能不依赖于RET的参与。因此，在不同的非小细胞肺癌细胞株中ARTN的表达水平并不能预测它的致癌性水平的相对高低。

在大多数恶性肺部肿瘤中，ARTN和受体GFRα3被检测到都有不同程度的升高，包括类癌、腺癌、鳞状细胞癌（非小细胞癌的两个主要亚型）和小细胞癌。此外，GFRα3在人和老鼠的正常肺和支气管中被检测出来^[58, 59]^，RET也被证实在人的正常肺中表达。这些事实进一步说明在人的肺中存在着ARTN自分泌的功能性信号转导途径，过度地激活此信号转导途径可以促进肺癌的进展；或放大和增加大多数其它癌基因的表达，包括表皮生长因子受体等，已有报道存在于一些肺癌的亚型^[60]^。

#### 5.5.7 ARTN与肺癌的预防、治疗和预后

Oncomine数据库显示ARTN的表达与肺癌患者的吸烟行为有极强的相关性；因此，我们在同是鳞状细胞癌患者中检测到了ARTN表达具有巨大差异；然而在并不吸烟的患者中，ARTN的表达在各个非小细胞肺癌的亚型中仍然是升高的，比如腺癌，这是在非吸烟肺癌患者中检测到的主要的亚型；还有类癌和小细胞肺癌中ARTN的表达相比于正常的肺组织也是升高的。

在上调ARTN的表达移植模型中，将导致形成大量空泡多核肿瘤细胞，这些肿瘤细胞微观上观察很像巨细胞癌，这种情况预示着肿瘤将出现独特的快速生长，远处广泛的转移，术后伴随着高复发的风险，并表现出对现有化疗方案的不敏感和很差的预期寿命，相对于其它类型的非小细胞肺癌亚型来说^[61, 62]^。

ARTN介导了非小细胞肺癌的进展，原因可能归结于ARTN在肿瘤的微环境中所起的旁分泌作用^[63]^。调高ARTN的表达，将增加非小细胞肺癌的侵袭性和转移性，同时也增加了TGFB1的表达。在原发性肺癌中，TGFB1表达的升高常常伴随着转移性的增加，这也提示肿瘤的高复发率和高死亡率^[52, 64]^。

已有研究证实在非小细胞肺癌细胞中ARTN的致癌性是通过升高BCL2的表达来实现的。在各种人类恶性肿瘤细胞中BCL2表现出增进细胞增殖和提高细胞存活率的作用，包括肺癌^[65, 66]^。在活体中ARTN的升高很可能是促进了BCL2的高表达，由此产生了一个致癌优势。增加BCL2的表达应先提高ARTN的表达，这些在人乳腺细胞和子宫内膜细胞中已被观察到^[49, 63, 67]^，抑制ARTN的表达用于降低非小细胞肺癌细胞的致癌性是可以期待的。

直到现在，还没有观测到BCL2的表达与非小细胞肺癌化疗敏感性存在相关性。尽管如此，BCL2似乎参与到人肺癌细胞株对化疗药物耐受的过程之中^[68-70]^，联合应用ABT-737（BCL2家族的药理抑制剂），在非小细胞肺癌细胞株中增强了紫杉醇和吉非替尼的细胞毒性^[71, 72]^。ABT-737和ABT-263不仅作为一个独立因素提供良好的临床前效能，而且明显地增进了人类小细胞肺癌和非小细胞肺癌细胞株的凋亡，并且增强了从这些细胞衍生的异形肿瘤对放疗和化疗的敏感性，使其退化消亡^[73]^。当前，ABT-263正在小细胞肺癌的患者中进行临床试验^[73]^。鉴于ARTN对紫杉醇有抵抗优势，因此抑制ARTN的表达用于提高非小细胞肺癌对化学的敏感性是可以期待的。

## 6 展望

ARTN的结构、分布、信号转导途径及对恶性肿瘤的发生、发展的作用具有其独特的一面。在治疗相关疾病的临床应用中可能较其它家族成员具有更好的应用前景。随着人类基因组计划的研究进展，遗传信息数据不断丰富，ARTN的功能表达的生理机制将被进一步明确，这将有助于为神经系统疾病，恶性肿瘤的靶向治疗提供理论依据，从而为神经系统疾病和恶性肿瘤的基因治疗及药物开发奠定基础。
